# Higher concentration of P7C3 than required for neuroprotection suppresses renal cell carcinoma growth and metastasis

**DOI:** 10.7150/jca.90439

**Published:** 2024-01-01

**Authors:** Ge Shu, Wenjin Chen, Chenchen Huang, Hui Shan, Jing Ye, Jianfa Li, Yaoting Gui

**Affiliations:** 1 Shenzhen Key Laboratory of Male Reproductive Medicine and Genetics, Institute of Urology, Peking University Shenzhen Hospital, Shenzhen, Guangdong, China.; 2 Department of Urology, Beijing Friendship Hospital, Capital Medical University, Beijing, China.; 3 Department of Neurosurgery, Peking University Shenzhen Hospital, Shenzhen Peking University-The Hong Kong University of Science and Technology Medical Center, Shenzhen, Guangdong Province, China.; 4 Department of Urology, Peking University First Hospital, Beijing, China.; 5 Institute of Precision Medicine, Peking University Shenzhen Hospital, Shenzhen, China.; 6 Department of Urology, Shanghai General Hospital, Shanghai Jiao Tong University School of Medicine, Shanghai, China.

**Keywords:** P7C3, renal cell carcinoma, RRM2, cGAS-STING, BAX

## Abstract

**Background:** P7C3 is a novel compound that has been widely applied in neurodegenerative diseases and nerve injury repair. Here, we show that higher concentrations of P7C3 than are required for in vivo neuroprotection have the novel function of suppressing renal cell carcinoma (RCC) proliferation and metastasis.

**Methods:** Colony formation, CCK-8 and EdU assay were applied to evaluate RCC cell proliferation. Wound healing and transwell assay were used to measure RCC cell migration and invasion. Flow cytometry assay was employed to detect RCC cell apoptosis and cell cycle. qRT-PCR assay was carried out to measure ribonucleotide reductase subunit M2 (RRM2) mRNA expression level, while western blot assay was utilized to detect the expression level of target proteins. RCC cell growth in vivo was determined by xenografts in mice.

**Results:** We observed that high concentrations of P7C3 could restrain the proliferation and metastasis of RCC cells and promote cell apoptosis. Mechanistically, this new effect of higher dose of P7C3 was associated with reduced expression of RRM2, and the beneficial efficacy of P7C3 in RCC was blocked when suppression of RRM2 was prevented. When RRM2 suppression was permitted, the cGAS-STING pathway was activated by virtue of RRM2/Bcl-2/Bax signaling. Lastly, intraperitoneal injection of this high level of P7C3 in mice potently inhibited tumor growth.

**Conclusion:** In conclusion, we show here that P7C3 that exerts an anti-cancer effect in RCC. Our study indicated that P7C3 might act as a novel drug for RCC in the future. The regulatory signal pathway RRM2/Bcl-2/BAX/cGAS-STING might present novel insight to the potential mechanism of RCC development.

## 1. Introduction

Renal cell carcinoma (RCC) is the second most lethal urologic tumor. It was estimated that there were 400,000 new cases of renal cancer worldwide and 175,000 people died from this disease 1. Partial nephrectomy is suitable for those patients with earlier stage renal cancer and their 5-year survival rate reached to 90% 2. However, approximately 30% of renal cancer patient were diagnosed as an advanced stage at their first exanimation 3. Despite aggressive therapy were given, it is inevitable that high rates of recurrence and metastasis, causing extremely low 5-year survival rate 4. Hence, it is imperative to investigate novel medicine to fight against renal cancer.

P7C3, a small molecule, was first discovered in 2010 that could efficiently cross the blood-brain barrier 5. As a neuroprotective compound, P7C3 has a good effect on neurodegenerative diseases and nerve injury repair 6. It was shown to protect newborn neurons and promote neurogenesis in adult mice. P7C3 can maintain the survival of nerve cell via activating nicotinamide phosphoribosyltransferase (NAMPT) activity and recovering the level of cellular nicotinamide adenine dinucleotide (NAD+) 7, 8. Several studies suggested that the derivatives of P7C3 (P7C3-A20 and P7C3-S243) could protect neuroprotective activity in animal models of Parkinson's disease and amyotrophic lateral sclerosis 9-13. Recent study has showed that P7C3 suppressed the growth and metastasis of glioma via inhibiting PGK1 expression and blocking the process of aerobic glycolysis 14. However, the role of P7C3 in renal cancer remains unclear.

In our study, we discovered that P7C3 could restrained the proliferation and metastasis of RCC cells and promoted cell apoptosis. Mechanically, P7C3 directly targeted on ribonucleotide reductase subunit M2 (RRM2) and suppressed the mRNA and protein expression of RRM2. RRM2 is the subunit of ribonucleotide reductase, getting involved in the production of deoxynucleic acid and nucleotide metabolism. Numerous studies suggest that RRM2 was dysregulated in various cancers, promoting the progression of cancers, such as renal cancer, liver cancer, lung cancer, breast cancer, colon cancer, ovarian cancer and bladder cancer 15-21. In renal cancer, RRM2 was dramatically overexpressed in tumor tissue, causing poor prognosis of cancer patients 22. OSAKO et al. discovered that RRM2 promoted RCC cell proliferation and impaired cell apoptosis in vitro 23. Xiong et al. found that RRM2 expression was dramatically increased in sunitinib-resistant RCC patient tissues and cells. RRM2 could enhance the sunitinib-resistant of RCC cells via stabilizing ANXA1 protein to activate PI3K/AKT signal pathway 24. However, we observed that RRM2 could accelerate the progression of RCC via suppressing the activity of Bcl-2/BAX/cGAS-STING signal pathway. In general, our study first reports the role of P7C3 in RCC progression in vitro and in vivo, expounding the molecular mechanism of P7C3 on the anti-cancer effects. These findings may provide a novel method to fight against renal cancer.

## 2. Material and methods

### 2.1 Cell lines

Human RCC lines Caki-1 and 786-O were provided by American Type Culture Collection (ATCC), USA. All cells included in this study were grown in DMEM medium (Gibco, Carlsbad, CA, USA) mixed with 10% fetal bovine serum (FBS) (Gibco, South America) and 1% antibiotics. All cells were cultured in a humidified incubator containing 5% CO2 at 37 °C.

### 2.2 Quantitative real-time PCR assay

Total RNA from RCC cell lines was extracted by utilizing TRIzol reagent. Then, a reverse transcription kit with a gDNA remover (TAKARA, JAPAN) was applied to reverse total RNAs. Quantification of mRNA was using by PrimeScript One Step RT-PCR Kit (TAKARA, JAPAN). The reaction of quantitative real-time PCR assay was carried out by a Roche LightCycler® 480II PCR instrument (Basel, Switzerland). GAPDH was used as an internal control. 2^-ΔΔCT^ method was applied to calculate the relative RNA expression levels.

### 2.3 Cell transfection

The coding sequence of RRM2 was cloned into pcDNA3.1 vector. pcDNA3.1-negative control (NC) and pcDNA3.1-RRM2 were purchased from GenePharma (Suzhou, China). The plasmid of pcDNA3.1-RRM2 was extracted by using the Plasmid Maxi Kit (OMEGA, USA). The plasmid of pcDNA3.1-RRM2 or pcDNA3.1-NC were transfected into Caki-1 and 786-O cells by utilizing Lipofectamine 3000 (Invitrogen, USA) according to the instructions.

### 2.4 Cell proliferation assay

A colony-formation assay and an EdU assay were employed to detect the proliferation of RCC cells. For the colony-formation assay, the treated cells were seeded into a 6-well plate at a density of 1000 cells per well and cultured in the incubator for 14 days. Then, the treated cells were washed by PBS solution, fixed by 4% paraformaldehyde and stained with 0.1% crystal violet. The stained cells were imaged and washed with 33% glacial acetic acid. The absorbance of scrubbing solution was detected by using a microplate reader at 550 nm. For EdU assay, treated cells were seeded in a 12-well plate covered with carry with glass. An EdU assay kit (Ribobio, Guangzhou, China) was applied to performed the EdU assay. Cells adhered to carry with glass were photographed by using a microscope (Leica, Germany) under a fluorescence mode. The proportion of EdU positive cells to DAPI-stained cells was equal to the proliferation rate of treated cells.

### 2.5 Cell migration and invasion assays

A scratch test assay and a transwell assay was carried out to measure the migratory ability of RCC cells. For scratch test assay, RCC cells cultured in a 6-well plate and grown to 100% confluence were exposed to 30 μM of P7C3. A 200 ul pipette tip was applied to generate a clear scar in the cell layer. Images of cells were taken by a microscope (Leica, Germany) at 0 h and 24 h. For the transwell migration assay, RCC cells with 100 ul of serum-free medium were added in the upper chamber, and the lower chamber was stuffed with 500 ul of DMEM medium with 10% FBS. After incubation for 48 h, RCC cells crossed over the chamber were 4% paraformaldehyde, stained with 0.1% crystal violet and imaged. The stained cells were washed by 33% glacial acetic acid, and the absorbance of scrubbing solution were measured by a microplate reader at 550 nm. The experimental method of the transwell invasion assay was identified with that of the transwell migration assay. However, the upper chambers were covered with diluted Matrigel (BD Biosciences, USA).

### 2.6 Cell cycle assay

RCC cells seeded in to a 6-well plate were exposed to 30 μM of P7C3. Cells were washed with PBS solution twice, fixed with 70% cold ethyl alcohol and incubated at 4℃ overnight. Each sample was mixed with RNase enzyme and incubated at room temperature for half an hour. Then, each sample was incubated with PI kit. Finally, cell cycle detection was determined by a flow cytometry (Beckman, CA, USA).

### 2.7 Cell apoptosis assay

RCC cells seeded in to a 6-well plate were exposed 30 μM of P7C3. Cells were harvested after digestion of pancreatin without EDTA. Then, cells were resuspended after washing with PBS solution. Finally, all samples were added with Annexin V and PI kit. Cell apoptosis was determined by a flow cytometry (Beckman, CA, USA).

### 2.8 RNA-sequence assay

The data set included Caki1 and 786-O cell lines treated with 30 μM of P7C3 or PBS solution in thrice replicates. 1ug total RNA was applied for cDNA libraries using cDNA-PCR Sequencing Kit (SQK-LSK110+EXP-PCB096). Transcripts were validated against known reference transcript annotations with gffcompare. Gene function was annotated based on NCBI, Protein family, KOG/COG/eggNOG, KEGG and GO databases. Differential expression analysis of two conditions/groups was performed using the DESeq2 R package. Genes with a FDR < 0.01 and foldchange ≥ 2 found by DESeq2 were regarded as differentially expressed.

### 2.9 Tandem mass tag proteomics analysis

Total protein from Caki-1 and 786-O cell lines were treated with 30 μM of P7C3 or PBS solution were extracted and determined by using a BCA kit (Thermo Fisher Scientific, USA). Total protein was separated by 12.5% SDS-PAGE gel. Filter-aided sample preparation was performed by using SDT buffer and UA buffer. The produced peptides were collected as a filtrate. Then, the peptide mixture was labelled by utilizing a TMT kit (Thermo Fisher Scientific, USA). Agilent 1260 Infinity II HPLC system was applied to segregate the labeled peptides. Each component of labeled peptides was analyzed by using nano LC-MS/MS system. LC-MS/MS analysis was performed by using a Q Exactive plus mass Spectrometer (Thermo Fisher Scientific, USA) and Easy nLC (Thermo Fisher Scientific, USA) for one hour and a half. Mascot engine embedded into Proteome Discoverer 2.2 (Thermo Fisher Scientific, USA) was applied to analyze the MS/MS raw data.

### 2.10 Western blotting assay

Total protein from RCC cells was extracted using RIPA lysis buffer (Beyotime, China). Total protein was boiled and quantified by using BCA protein assay kit (Abcam, UK). Equal amounts of protein were separated by 10% SDS-PAGE gels and transferred onto 0.45 μm PVDF membranes (Millipore, USA). The membranes were blocked by 5% skim milk in TBST solution and washed with TBST solution three times. Then, the membranes were incubated with a primary antibody at 4 °C for 12 h, followed by a secondary antibody at room temperature for 2h. At last, the membranes were soaked in DAB Horseradish Peroxidase Color Development Kit (Beyotime, China). All membranes were exposed to a BioSpectrum 600 Imaging System (UVP, USA).

### 2.11 Immunofluorescence assay

The transfected cells were seeded into 24-well plates covered with glass coverslips for a day. The cells were washed by PBS solution and fixed with 4% of paraformaldehyde. After incubation of skim milk, the glass coverslips were incubated with primary antibodies against RRM2 overnight. Finally, images were captured by using a fluorescence microscope after incubation of fluorescent secondary antibody.

### 2.12 Xenografts in mice

The growth of RCC cells in vivo was determined by Xenografts in mice. The tumor transplantation experiment was examined and approved by the Ethics Committee of Peking University Shenzhen Hospital. All animal care and experimental procedures were in line with the requirement of animal ethics. 12 4-week-old male BALB/c nude mice were randomized to NC group and P7C3-treated group. Approximately 5×10^6^ 786-O cells were injected into the backs of nude mice. Until the size of xenograft tumors reached to 100mm^3^, mice in P7C3-treated group was peritoneal injection with 10 mg/kg/body P7C3 daily, while negative control group was given intraperitoneal injections of normal saline daily. The size of all xenograft tumors was measured by dividing rule per week on day 14 after beginning P7C3 treatment. All nude mice were sacrificed by carbon dioxide after the injection for 4 weeks, and the xenograft tumors were weighed.

### 2.13 Statistical analysis

All date from three times independent replication experiment were analyzed by using SPSS 20.0 and GraphPad Prism. All date was presented as Mean ± S.E. Student's t-test was applied to analyze the difference in two groups. ANOVA test was employed to analyze the results of CCK-8 assay. P < 0.05 was considered statistically significant in all results.

## 3. Results

### 3.1 P7C3 suppressed the proliferation of RCC cells

**Figure 1:**
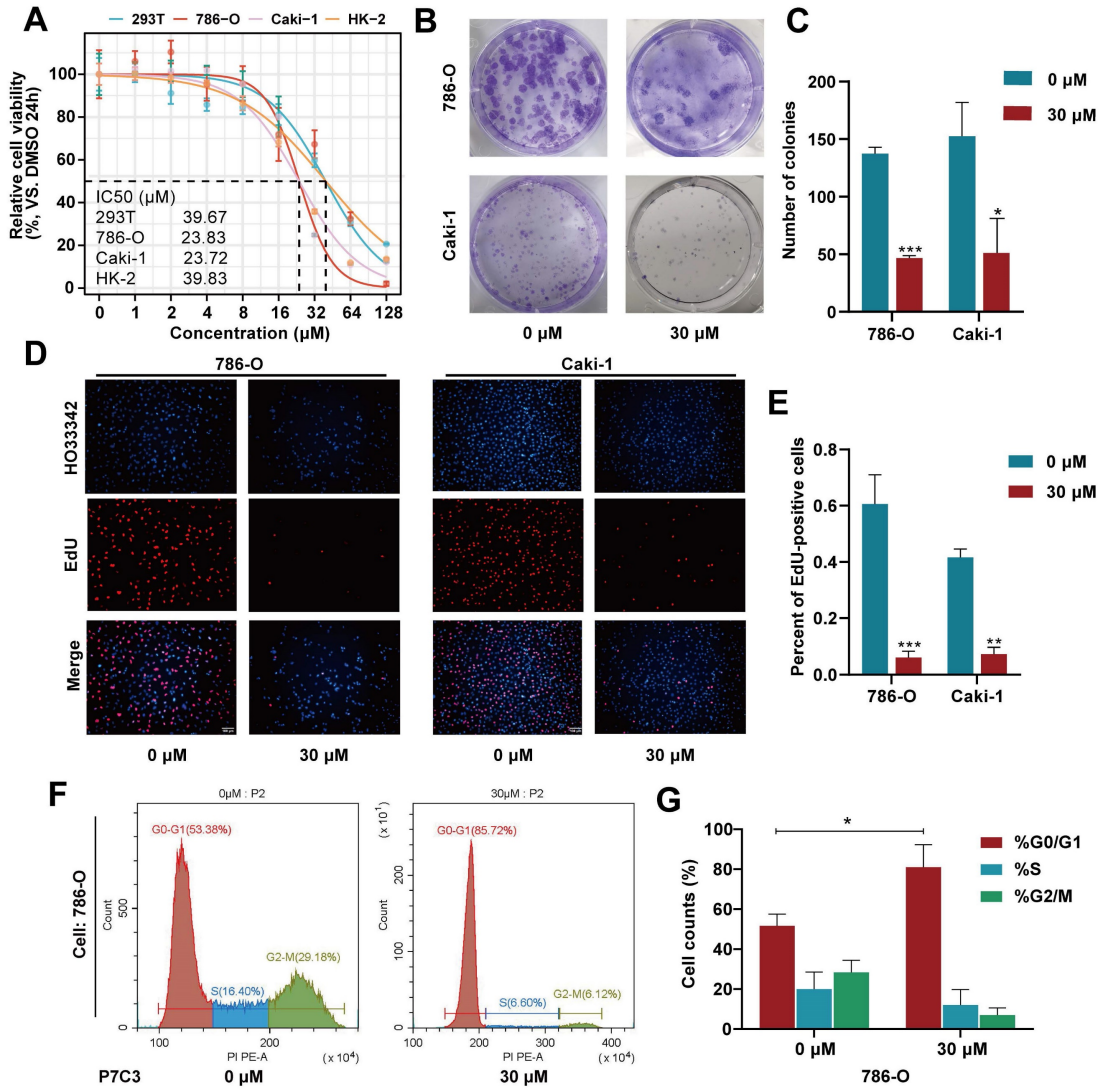
P7C3 suppress the proliferation of RCC cells. (A) The IC50 value of 293T, 786-O, Caki-1 and HK-2 cells treated with P7C3. (B and C) The colony numbers of 786-O and Caki-1 cells treated with 30 μM of P7C3 or PBS solution. (D and E) The proliferative rate of 786-O and Caki-1 cells treated with 30 μM of P7C3 or PBS solution. (F and G) Cell cycle of 786-O and Caki-1 cells treated with 30 μM of P7C3 or PBS solution. *p < 0.05, **p < 0.01, ***p < 0.001.

As shown in Fig. 1A, the IC50 value of P7C3 in 293-T, 786-O, Caki-1 and HK-2 was 39.67μM, 23.83μM, 23.72μM and 39.83μM, respectively (Fig. 1A). Colony formation assay presented that the colony numbers of 786-O and Caki-1 cells decreased dramatically while cells were exposed to 30 μM of P7C3 (Fig. 1B and C). In addition, the results of EdU assay were similar to that of Colony formation assay. EDU-positive cells were significantly impaired while 786-O and Caki-1 cells were exposed to 30 μM of P7C3 (Fig. 1D and E). Cell cycle assay showed that P7C3 caused a reduction of S phase (from 16.4% to 6.6% in 786-O cell) and an decrease of G2/M phase (from 29.18% to 6.12% in 786-O cell) (Fig. 1F and G). These results indicating that P7C3 could suppressed the proliferation of RCC cells.

### 3.2 P7C3 suppressed the metastasis and promoted the apoptosis of RCC cells

**Figure 2:**
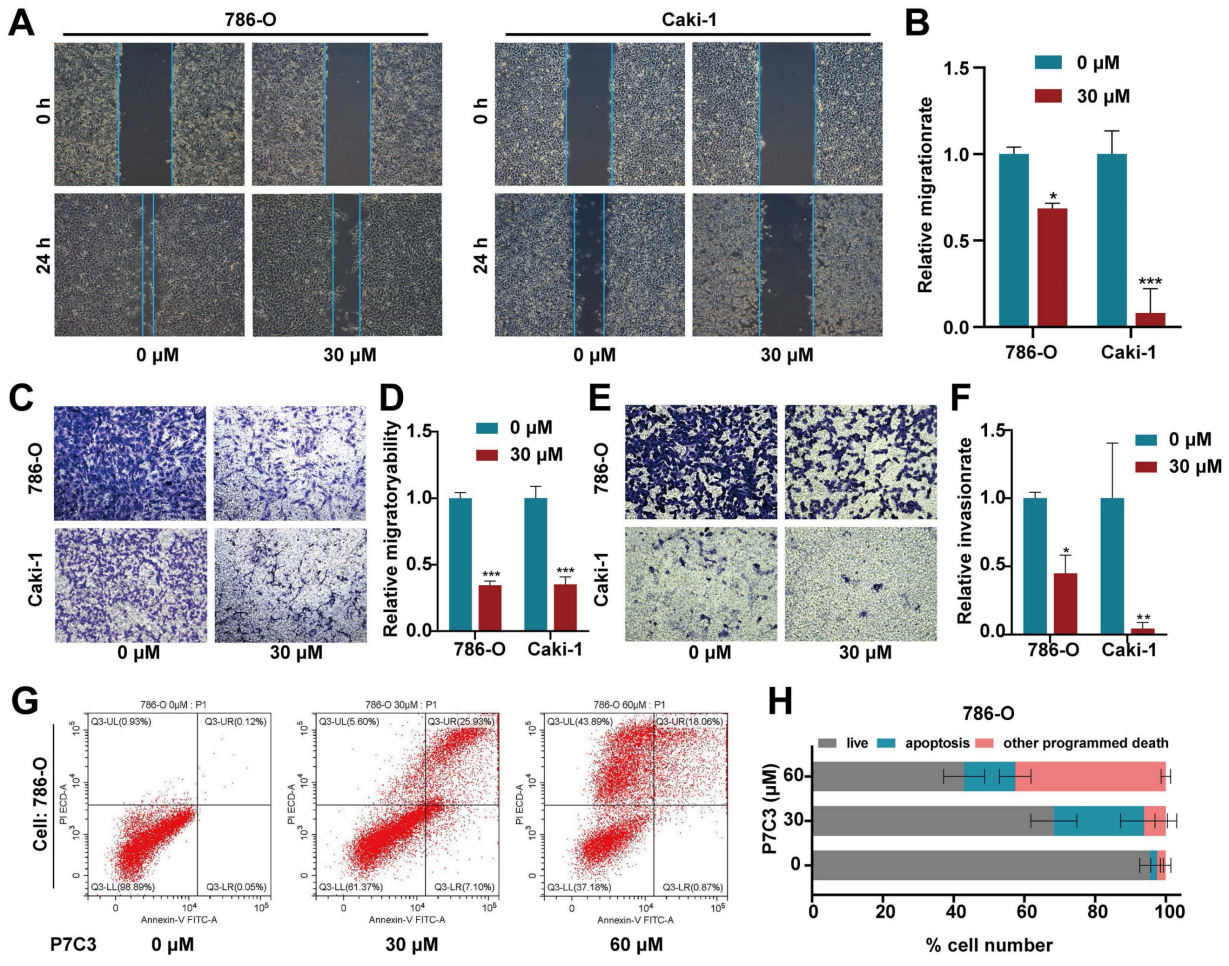
P7C3 suppress RCC metastasis and promote cell apoptosis. (A and B) The migration distance of 786-O and Caki-1 cells treated with 30 μM of P7C3 or PBS solution. (C and D) P7C3 restrained the migration of 786-O and Caki-1 cells. (E and F) P7C3 restrained the invasion of 786-O and Caki-1 cells. (G and H) The apoptosis rate of 786-O and Caki-1 cells treated with PBS solution, 30 μM of P7C3 and 60 μM of P7C3. *p < 0.05, **p < 0.01, ***p < 0.001.

The scratch assay and transwell assay were conducted to detect the migration and invasion of RCC cell. The results of scratch assay showed that P7C3 significantly impaired the migration of Caki-1 and 786-O cells (Fig. 2A and B). Transwell migration assay demonstrated that the number of cells crossed the chamber in P7C3-treated group were dramatically less than that in negative control group (Fig. 2C and D). Transwell invasion assay presented that P7C3 significantly impaired the invasion of RCC cells (Fig. 2E and F). Flow Cytometry analysis was applied to measure cell apoptosis. As shown in Fig. 2G and H, 30 μM of P7C3 obviously facilitated the apoptosis of RCC cells, whereas 60 μM of P7C3 dramatically promoted the death and apoptosis of RCC cells. These results demonstrated that P7C3 restrained the migration and invasion of RCC cells and accelerated the apoptosis of RCC cells.

### 3.3 P7C3-mediated suppression of RCC is associated with reduced expression of RRM2

**Figure 3:**
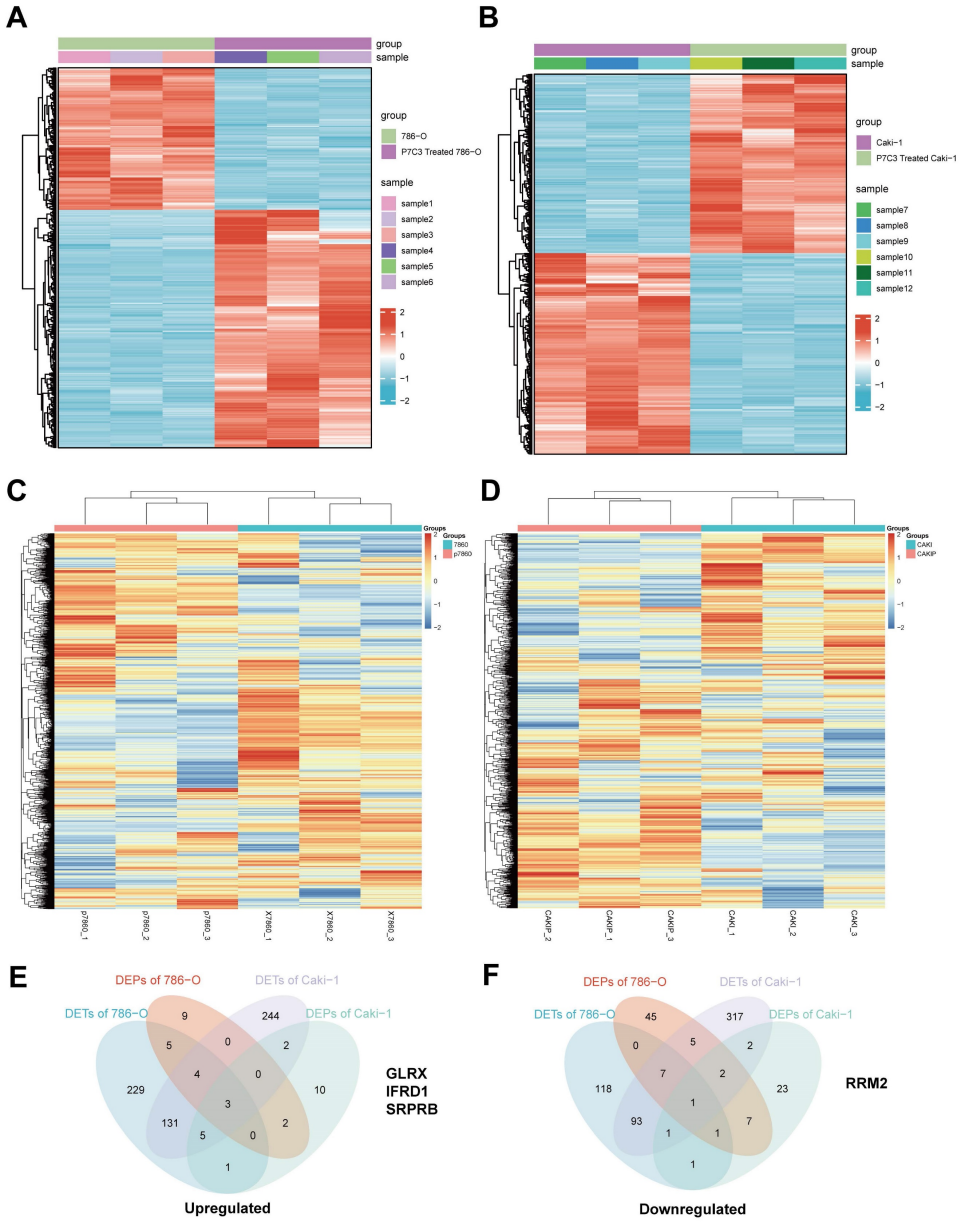
Differentially expressed genes and proteins in RCC cells and RCC cells treated with 30 μM of P7C3. (A) The cluster heat maps show differentially expressed genes in 786-O cell and 786-O cell treated with 30 μM of P7C3. (B) The cluster heat maps show differentially expressed genes in Caki-1 cell and Caki-1 cell treated with 30 μM of P7C3. (C) The cluster heat maps show differentially expressed proteins in 786-O cell and 786-O cell treated with 30 μM of P7C3. (D) The cluster heat maps show differentially expressed proteins in Caki-1 cell and Caki-1 cell treated with 30 μM of P7C3. (E) The Venn diagram exhibiting the overlap between up-regulated gene cluster and protein cluster heat maps. (F) The Venn diagram exhibiting the overlap between down-regulated gene cluster and protein cluster heat maps.

**Figure 4:**
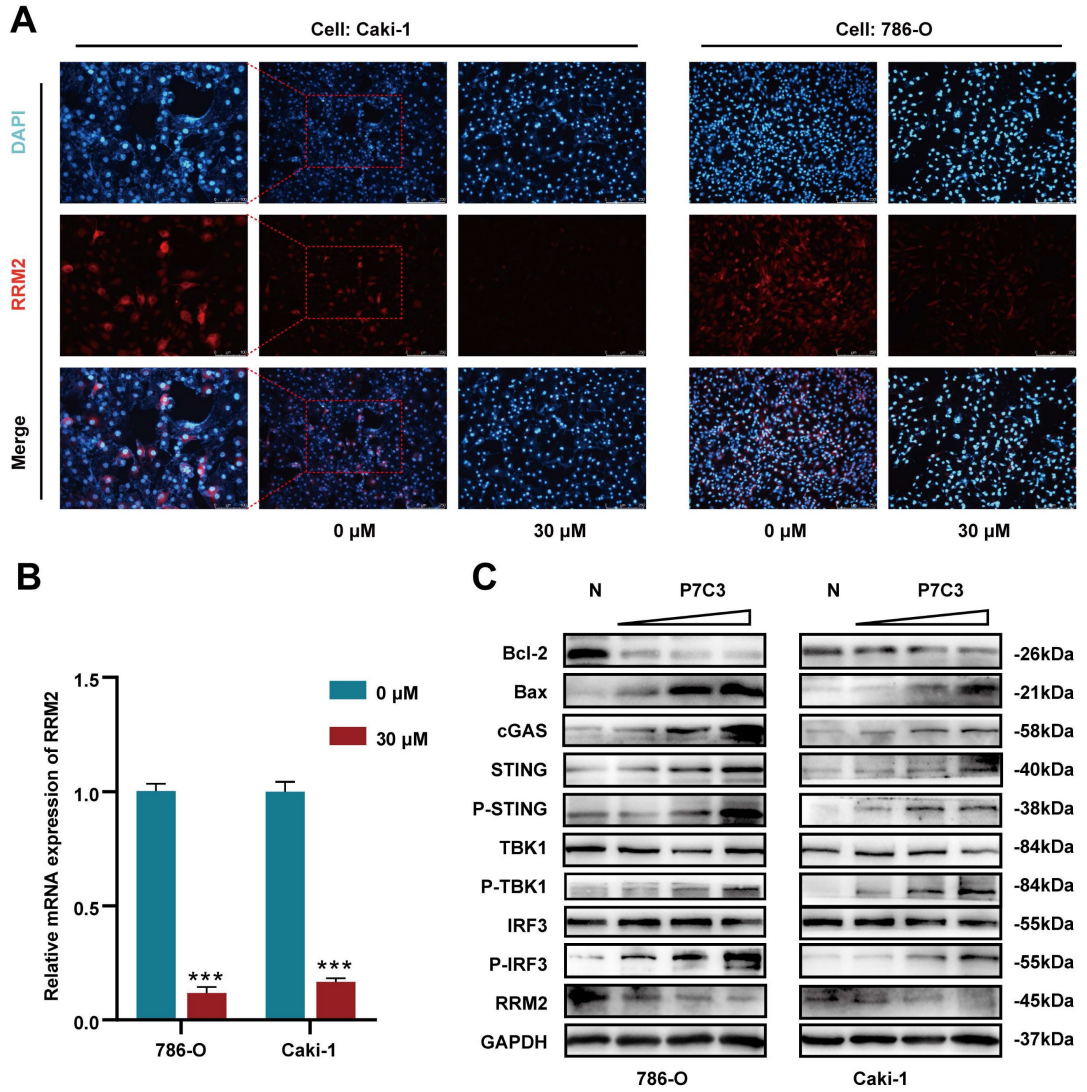
P7C3 activates cGAS-STING signal pathway. (A) The protein expression of RRM2 in 786-O and Caki-1 treated with 30 μM of P7C3 or PBS solution. (B) The Mrna expression of RRM2 in 786-O and Caki-1 treated with 30 μM of P7C3 or PBS solution. (C) Western blot analysis of RRM2, Bcl-2, BAX, cGAS, STING, p-STING, TBK1, p-TBK1, IRF3, p-IRF3 in 786-O and Caki-1 cells treated with different concentration of P7C3 or PBS solution. *p < 0.05, **p < 0.01, ***p < 0.001.

To elucidate the molecular mechanism of anticancer effects mediated by PC73, we applied high throughput sequencing and observed that 507 transcripts are overexpressed in 786-O cell treated with P7C3, while 303 transcripts are down-regulated (Fig. 3A). Meanwhile, 530 transcripts are overexpressed in Caki-1 cell treated with P7C3, while 303 transcripts are down-regulated (Fig. 3B). 23 proteins are overexpressed in 786-O cell treated with P7C3, while 74 proteins are down-regulated (Fig. 3C). 23 proteins are overexpressed in Caki-1 cell treated with P7C3, while 38 proteins are down-regulated (Fig. 3D). 4 genes are the complication of the results of RNA-seq and proteomics data, including GLRX, IFRD1, SRPRB, and RRM2 (Fig. 3E and F). RRM2 expression was significantly decreased when cells were treated with P7C3, while the expression level of GLRX, IFRD1, SRPRB and were augmented obviously upon P7C3 treatment. Further experiments demonstrated that P7C3 not only suppressed RRM2 mRNA expression, but also impaired RRM2 protein expression (Fig. 4A and B). Previous study suggested that knockdown of RRM2 cause cell apoptosis via accelerating the degradation of Bcl-2 protein 25. Another study showed that Bcl-2 associated X protein (BAX) could activate the cGAS-STING pathway via inducing mitochondrial damage and mtDNA leakage to the cytosol 26. We suspected that P7C3 could activate the cGAS-STING pathway via regulating RRM2/Bcl-2/BAX signal pathway. As shown in Fig 4C, P7C3 suppressed RRM2 protein expression to inhibited Bcl-2 protein expression and increase BAX protein expression, which activating cGAS-STING pathway. In addition, P7C3 enhanced the phosphorylation of STING, TBK1 and IRF3. These results suggested that P7C3 could inhibited RRM2 expression to activate the cGAS-STING pathway.

### 3.4 Increased RRM2 expression reverses the restraining influence mediated by P7C3 inhibition on cell growth and metastasis

**Figure 5:**
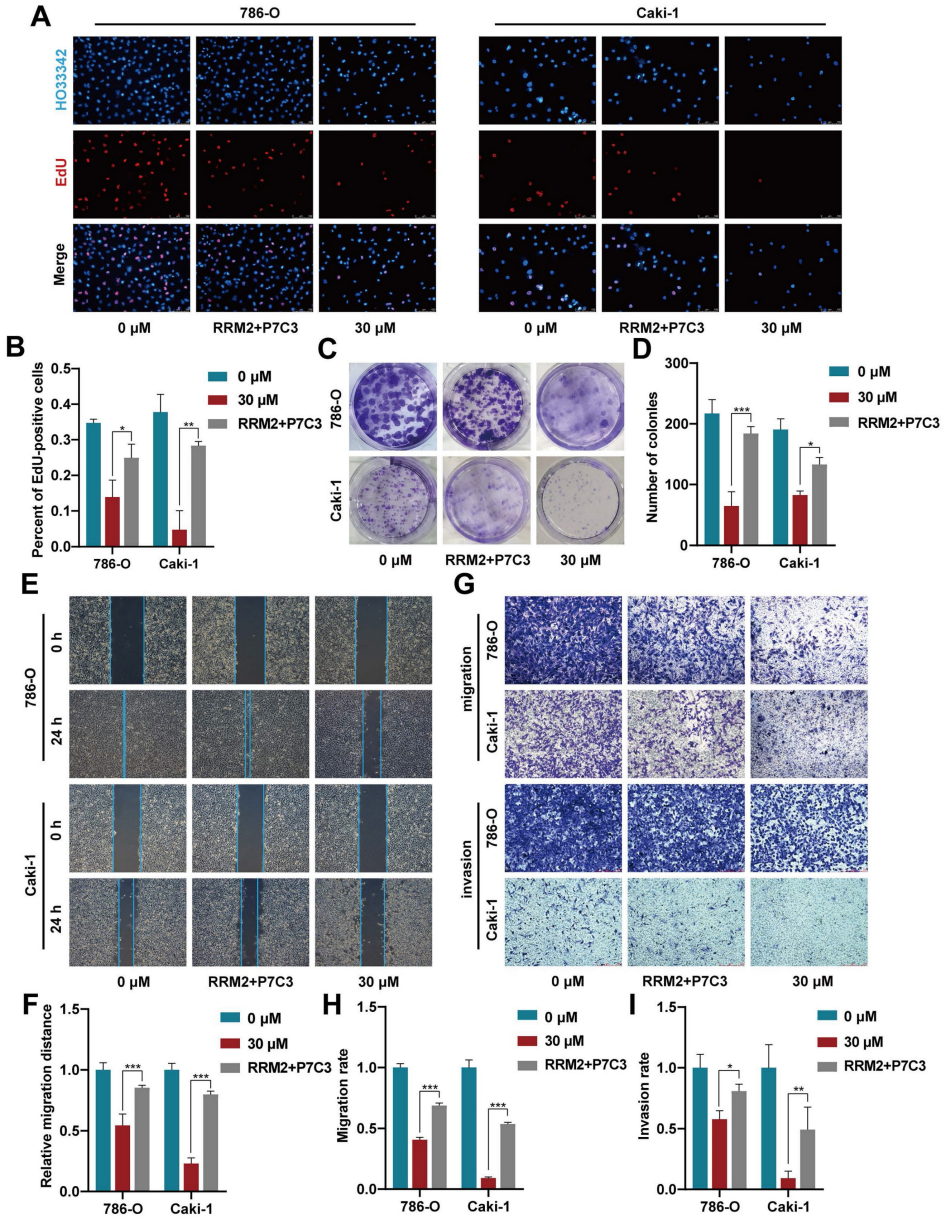
RRM2 overexpression reverses P7C3-induced reduce in cell growth and metastasis in RCC cells. (A-D) Augment of RRM2 reversed P7C3-induced proliferation suppression. (E-I) Augment of RRM2 reversed P7C3-induced migration and invasion suppression. *p < 0.05, **p < 0.01, ***p < 0.001.

To detect whether P7C3 exerts its anticancer effect by modulating RRM2 expression, rescue assay between P7C3 and RRM2 was carried out. EdU assay demonstrated that increased RRM2 expression obviously resisted the P7C3-induced proliferation inhibition in RCC cells (Fig. 5A and B). Colony formation assay presented that forced RRM2 expression significantly retrieved the colony numbers reduction mediated by P7C3 treatment in RCC cells (Fig. 5C and D). Wound-healing assay demonstrated that augmented RRM2 expression reversed RCC cell migration suppression induced by P7C3 (Fig. 5E and F). Finally, transwell migration and invasion assay showed that the inhibitory effect of P7C3 treatment on cell migration and invasion could be reversed while RRM2 expression was enhanced (Fig. 5G-I). These results indicated that overexpression of RRM2 reversed inhibitory effect of P7C3 treatment on cell growth and metastasis.

### 3.5 P7C3 restrained RCC growth in vivo

**Figure 6:**
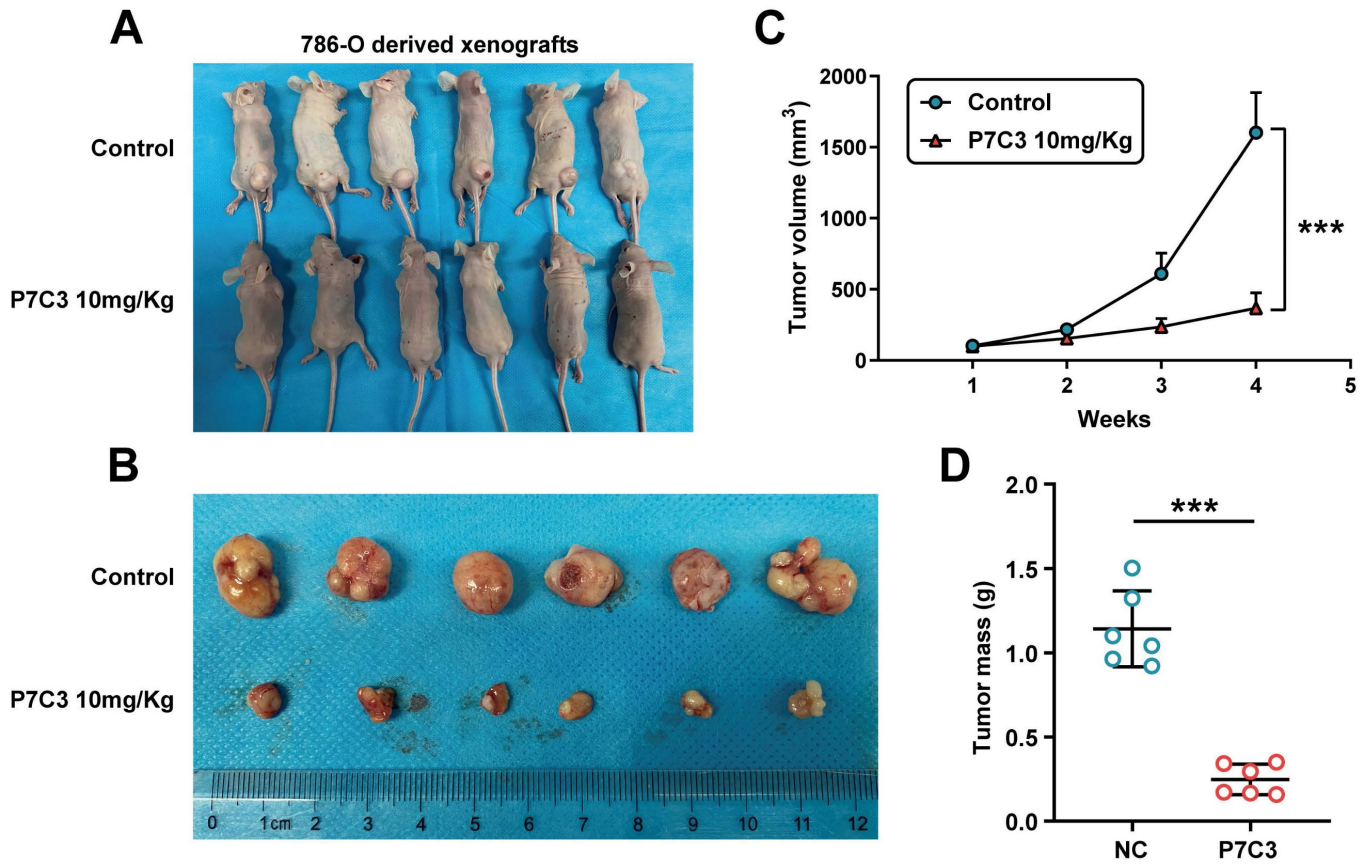
RRM2 suppress RCC cell growth in vivo. (A and B) The size of transplanted tumors treated with P7C3 were much smaller than negative control group. (C) The growth rate of transplanted tumors in P7C3-treated group and negative control group. (D) The quality of transplanted tumors in P7C3-treated group and negative control group. *p < 0.05, **p < 0.01, ***p < 0.001.

To investigate the role of P7C3 in RCC cell growth in vivo, 786-O cells were injected into the back of nude mice to produce a xenograft tumor model. Mice were peritoneal injection with P7C3 plus physiological saline was while tumors grew to 100 mm^3^ (Fig. 6A). In the xenograft tumor model, the transplanted tumors treated with P7C3 were much smaller than those treated with physiological saline (Fig. 6B). Apparently, PC73 inhibited the growth rate and weight of tumors (Fig. 6C and D). These results demonstrated that P7C3 inhibited the growth of RCC cells in vivo.

## 4. Discussion

Renal cell carcinoma is a common malignant tumor in the urinary system, accounting for 2%-3% of adult malignant tumors 1. RCC is occult, and there are no obvious symptoms in the early stage. However, approximately 25-30% of RCC patients have distant metastasis at the first time of diagnosis 27. Palliative treatment is applied for metastatic RCC patients which is not sensitive to radiotherapy and chemotherapy, with only 10% 5-year survival rate 28. In addition, about 18-23 % limited RCC patients developed to distant metastasis after surgery, causing poor prognosis 29. Hence, it is urgent to explore novel method to fight against RCC.

P7C3, a novel compound, containing screening Pool #7 (P7) and aminopropyl carbazole Compound #3 (C3) was discovered to enhance the survival of newborn neurons, instead of accelerating the proliferation rate 11. P7C3 compounds have been shown to be safe and neuroprotective in nonhuman primates 30. Numerous studies showed that P7C3 compounds contributes to the treatment of traumatic brain injury 6, 9, 31-35, Alzheimer's disease 36, amyotrophic lateral sclerosis 37, Parkinson's disease 10, 38 and stroke 39, 40.

In a zebrafish model of retinal degeneration, P7C3 effectually restrained the apoptosis of retinal 41. In preclinical models of depression and anxiety, P7C3 could suppress the progression of hippocampal cell death 37, 42. Wallker et al. discovered that the P7C3 compounds play a role in antidepressant efficacy in mice via enhancing hippocampal neurogenesis 43. In addition, they also discovered that P7C3-A20 contributed to the treatment of post-traumatic stress associated depression and anxiety, as well as prenatal maternal stress 44-46. In preclinical models of peripheral neuropathy caused by chemotherapy and Alzheimer's disease, P7C3 could reverse the progress of neurological dysfunction and nerve cell death 36, 47, 48. Most important of all, the potency, stability and safety of P7C3 were proved by several studies 32. Recently, the anti-cancer effects of P7C3 were proved in glioma, opening up a new vision of P7C3 application.

In our study, we observed that high concentration of P7C3 could inhibit the growth of RCC cells in vitro and in vivo. Vazquez-Rosa et al. showed that low concentration of P7C3-A20 (0.3, 1, 3, and 5 μM) could recover the blood-brain barrier, suppress chronic neurodegeneration and restore cognition in mice with traumatic brain injury, while these concentrations of P7C3 have no effect in the RCC culture system. We suspected that higher doses of P7C3 than are needed to protect nerve cells are able to shut down cancer cell proliferation. Secondly, the metabolic rate of cancer cells is different from that in nerve cells. Thirdly, low concentrations of P7C3 may be easily cross the blood-brain barrier instead of high concentrations of P7C3. We also observed that P7C3 significantly restrained RCC metastasis and enhance cell apoptosis. However, 30 μM of P7C3 caused 25.93% of cell apoptosis and 5.6% of cell death, while 60 μM of P7C3 caused 18.06% of cell apoptosis and 43.89% of cell death. We suspected that high concentration of P7C3 might trigger other mechanisms of procedural death. Mechanistically, P7C3 could suppress the translation of RRM2. Numerous studies suggest that RRM2 expression was dramatically increased in various cancer tissues, causing unfavorable prognosis to these patients 15. In vitro and in vivo assay demonstrated that RRM2 got involved in the progression of cancers, such as cell proliferation, cell apoptosis, cell metastasis, chemoresistance, energy metabolism and immune resistance 49, 50. In melanoma, RRM2 could enhance melanoma grow and promote cellular senescence 51. In breast cancer, RRM2 accelerated cell migration and invasion via regulating epithelial-mesenchymal transition (EMT) process 52. In head and neck and lung cancers, knockdown of RRM2 caused cell apoptosis via accelerating the degradation of Bcl-2 25. In lung adenocarcinoma (LUAD), high expression of RRM2 is regarded as a potential independent risk factor and correlated with immune infiltration. Zhang et al. found that RRM2 could enhance the proliferation and metastasis of LUAD cells. In adddiotn, RRM2 could promote cell proliferation and suppress cell apoptosis via impairing the activity of cGAS/STING signaling pathway 53. The cyclic GMP-AMP synthase (cGAS)-stimulator of interferon genes (STING) pathway is regarded as an important innate immune pathway and activated by double-stranded DNA, promoting unique immune effector responses that can regulate different kinds of tumorigenesis, including cell growth, cell metastasis, chemotherapy resistance and immune escape 54-56. cGAS can produce cyclic GMP-AMP (cGAMP), a distinct mixed-linkage second-messenger member molecule, binding and activing STING. STING activation launch the downstream transcriptional progression including nuclear factor (NF)-κB and interferon (IFN) regulatory factor 3 (IRF3) to augment the expression of chemokines, proinflammatory cytokines and type I IFNs 57, 58. However, the regulatory pathway between RRM2 and cGAS/STING signaling pathway remains unclear. Recently, a study suggest that Bcl-2 associated X protein (BAX) could activate the cGAS-STING pathway via inducing mtDNA cytosolic leakage 59, 60. Therefore, we suspected that P7C3 might activate cGAS-STING via regulating Bcl-2/BAX pathway. Western blot assay suggested that P7C3 obviously impaired Bcl-2 protein expression and increased BAX protein expression, indicating that P7C3 could activate cGAS-STING signal pathway via regulating BAX expression. Further experimental results confirmed that P7C3 could activate cGAS-STING signal pathway via regulating RRM2/Bcl-2/BAX pathway. Finally, rescue assays demonstrated that P7C3 exert its anti-cancer effects via regulating RRM2 expression.

In conclusion, we identified the novel compound P7C3 that exerts an anti-cancer effect in RCC. Further experiments demonstrated that P7C3 suppressed RRM2 expression to disequilibrate the balance between Bcl-2 and BAX and impair the activity of cGAS-STING signal pathway, inhibiting the development of RCC. Our study indicated that P7C3 might act as a novel drug for RCC in the future. The regulatory signal pathway RRM2/Bcl-2/BAX/ cGAS-STING might present novel insight to the potential mechanism of RCC development.
